# The ATLAS/NOA-29 study protocol: a phase III randomized controlled trial of anterior temporal lobectomy versus gross-total resection in newly-diagnosed temporal lobe glioblastoma

**DOI:** 10.1186/s12885-025-13682-3

**Published:** 2025-02-20

**Authors:** Matthias Schneider, Anna-Laura Potthoff, Yahya Ahmadipour, Valeri Borger, Hans Clusmann, Stephanie E. Combs, Marcus Czabanka, Lasse Dührsen, Nima Etminan, Thomas M. Freiman, Ruediger Gerlach, Florian Gessler, Frank A. Giordano, Eleni Gkika, Roland Goldbrunner, Erdem Güresir, Hussam Hamou, Peter Hau, Sebastian Ille, Max Jägersberg, Naureen Keric, Maryam Khaleghi-Ghadiri, Ralph König, Jürgen Konczalla, Harald Krenzlin, Sandro Krieg, Aaron Lawson McLean, Julian P. Layer, Jens Lehmberg, Vesna Malinova, Bernhard Meyer, Hanno S. Meyer, Dorothea Miller, Oliver Müller, Christian Musahl, Barbara E. F. Pregler, Ali Rashidi, Florian Ringel, Constantin Roder, Karl Rössler, Veit Rohde, I. Erol Sandalcioglu, Niklas Schäfer, Christina Schaub, Nils Ole Schmidt, Gerrit A. Schubert, Clemens Seidel, Corinna Seliger, Christian Senft, Julia Shawarba, Joachim Steinbach, Veit Stöcklein, Walter Stummer, Ulrich Sure, Ghazaleh Tabatabai, Marcos Tatagiba, Niklas Thon, Marco Timmer, Johannes Wach, Arthur Wagner, Christian Rainer Wirtz, Katharina Zeiler, Thomas Zeyen, Patrick Schuss, Rainer Surges, Christine Fuhrmann, Daniel Paech, Matthias Schmid, Yvonne Borck, Torsten Pietsch, Rafael Struck, Alexander Radbruch, Christoph Helmstaedter, Robert Németh, Ulrich Herrlinger, Hartmut Vatter

**Affiliations:** 1 Department of Neurosurgery, University Hospital Bonn, Venusberg Campus 1, Bonn, 53127 Germany; 2 Brain Tumor Translational Research Group, University Hospital Bonn, Bonn, Germany; 3 Department of Neurosurgery and Spine Surgery, University Hospital Essen, Essen, Germany; 4 Department of Neurosurgery, RWTH Aachen University Hospital, Aachen, Germany; 5 Department of Radiation Oncology, Klinikum Rechts Der Isar, Technical University of Munich (TUM), Munich, Germany; 6 Department of Neurosurgery, University Hospital Frankfurt, Frankfurt, Germany; 7 Department of Neurosurgery, University Medical Center Hamburg-Eppendorf, Hamburg, Germany; 8 Department of Neurosurgery, University Hospital Mannheim, Mannheim, Germany; 9 Department of Neurosurgery, University Medical Center Rostock, Rostock, Germany; 10 Department of Neurosurgery, Helios Kliniken, Erfurt, Germany; 11 Department of Radiation Oncology, University Hospital Mannheim, Mannheim, Germany; 12 Department of Radiation Oncology, University Hospital Bonn, Bonn, Germany; 13 Department of General Neurosurgery, Center of Neurosurgery, University of Cologne, Cologne, Germany; 14 Department of Neurosurgery, University Hospital Leipzig, Leipzig, Germany; 15 Department of Neurology and Wilhelm Sander-Therapy Unit, Regensburg University Medical Center, Regensburg, Germany; 16 Department of Neurosurgery, Heidelberg University Hospital, Heidelberg, Germany; 17 Department of Neurosurgery, University Medical Center Mainz, Mainz, Germany; 18 Department of Neurosurgery, University Medical Center Schleswig-Holstein/Lübeck, Lübeck, Germany; 19 Department of Neurosurgery, University Hospital of Münster, Münster, Germany; 20 Department of Neurosurgery, University of Ulm, Günzburg, Germany; 21 Department of Neurosurgery, Jena University Hospital, Jena, Germany; 22 Institute of Experimental Oncology, University Hospital Bonn, Bonn, Germany; 23 Department of Neurosurgery, München Klinik Bogenhausen, Munich, Germany; 24 Department of Neurosurgery, University Medical Center Göttingen, Göttingen, Germany; 25 Department of Neurosurgery, Klinikum Rechts Der Isar, Technical University of Munich (TUM), Munich, Germany; 26 Department of Neurosurgery, University Hospital Knappschaftskrankenhaus Bochum, Ruhr University Bochum, Bochum, Germany; 27 Neurosurgical Department, Dortmund Hospital, Dortmund, Germany; 28 Department of Neurosurgery, Kantonspital Aarau, Aarau, Switzerland; 29 Department of Neurosurgery, University Hospital Magdeburg, Magdeburg, Germany; 30 Department of Neurosurgery, Ludwig Maximilian University (LMU) Hospital, Munich, Germany; 31 Department of Neurosurgery, University Hospital Tübingen, Tübingen, Germany; 32 Department of Neurosurgery, Medical University of Vienna, Vienna, Austria; 33 Department of Neurooncology, Center of Neurology, University Hospital Bonn, Bonn, Germany; 34 Department of Neurology, University Hospital Knappschaftskrankenhaus Bochum, Ruhr University Bochum, Bochum, Germany; 35 Department of Neurosurgery, University Hospital Regensburg, Regensburg, Germany; 36 Department of Radiation Oncology, University Hospital Leipzig, Leipzig, Germany; 37 Dr. Senckenberg Institute of Neurooncology, University Hospital Frankfurt, Frankfurt, Germany; 38 Department of Neurology and Interdisciplinary Neuro-Oncology, Hertie Institute for Clinical Brain Research, University Hospital Tübingen, Tübingen, Germany; 39 Department of Neurosurgery, Unfallkrankenhaus Berlin, Berlin, Germany; 40 Department of Epileptology, University Hospital Bonn, Bonn, Germany; 41 Clinical Study Core Unit Bonn, Institute of Clinical Chemistry and Clinical Pharmacology, University Hospital Bonn, Bonn, Germany; 42 Department of Neuroradiology, University Hospital Bonn, Bonn, Germany; 43 Department of Radiology, Brigham and Women’s Hospital, Harvard Medical School, Boston, USA; 44 Institute for Medical Biometry, Informatics and Epidemiology, University Hospital Bonn, Bonn, Germany; 45 Department of Neuropathology, University Hospital Bonn, Bonn, Germany

**Keywords:** Anterior temporal lobectomy, Epilepsy surgery, Temporal lobe glioblastoma, Gross-total resection, Supramarginal resection

## Abstract

**Background:**

The discovery of cellular tumor networks in glioblastoma, with routes of malignant communication extending far beyond the detectable tumor margins, has highlighted the potential of supramarginal resection strategies. Retrospective data suggest that these approaches may improve long-term disease control. However, their application is limited by the proximity of critical brain regions and vasculature, posing challenges for validation in randomized trials. Anterior temporal lobectomy (ATL) is a standardized surgical procedure commonly performed in patients with pharmacoresistant temporal lobe epilepsy. Translating the ATL approach from epilepsy surgery to the neuro-oncological field may provide a model for investigating supramarginal resection in glioblastomas located in the anterior temporal lobe.

**Methods:**

The ATLAS/NOA-29 trial is a prospective, multicenter, multinational, phase III randomized controlled trial designed to compare ATL with standard gross-total resection (GTR) in patients with newly-diagnosed anterior temporal lobe glioblastoma. The primary endpoint is overall survival (OS), with superiority defined by significant improvements in OS and non-inferiority in the co-primary endpoint, quality of life (QoL; “global health” domain of the European organization for research and treatment of cancer (EORTC) QLQ-C30 questionnaire). Secondary endpoints include progression-free survival (PFS), seizure outcomes, neurocognitive performance, and the longitudinal assessment of six selected domains from the EORTC QLQ-C30 and BN20 questionnaires. Randomization will be performed intraoperatively upon receipt of the fresh frozen section result. A total of 178 patients will be randomized in a 1:1 ratio over a 3-year recruitment period and followed-up for a minimum of 3 years. The trial will be supervised by a Data Safety Monitoring Board, with an interim safety analysis planned after the recruitment of the 57th patient to assess potential differences in modified Rankin Scale (mRS) scores between the treatment arms 6 months after resection. Assuming a median improvement in OS from 17 to 27.5 months, the trial is powered at > 80% to detect OS differences with a two-sided log-rank test at a 5% significance level.

**Discussion:**

The ATLAS/NOA-29 trial aims to determine whether ATL provides superior outcomes at equal patients’ Qol compared to GTR in anterior temporal lobe glioblastoma, potentially establishing ATL as the surgical approach of choice for isolated temporal glioblastoma and redefining the standard of care for this patient population.

**Trial registration:**

German Clinical Trials Register (DRKS00035314), registered on October 18, 2024.

## Background

Glioblastoma is the most common and most malignant primary brain tumor in adults, with a median overall survival (mOS) of about 17 months [1]. Surgical resection remains a pivotal component of multimodal therapy, with the extent of resection constituting a key determinant of long-term outcomes [2]. Known for their highly invasive nature, glioblastoma cells are found far beyond the solid tumor mass [3], with postmortem studies even reporting their presence in the contralateral hemisphere [4]. Rather than existing as isolated entities, these cells establish interconnected and adaptable communication networks that infiltrate distant brain regions [5, 6]. This malignant connectivity is thought to play a crucial role in therapy resistance and long-term treatment failure, emphasizing the need for strategies that address the tumor’s extensive infiltrative growth pattern [7].

From a surgical perspective, supramarginal resection has gained increasing attention in recent years [8]. Unlike conventional gross-total resection (GTR), which focuses on removing the gadolinium-enhancing tumor core visible on magnetic resonance imaging (MRI) and the adjacent tissue of the near infiltration zone, supramarginal resection extends further into more distant infiltrative regions [9]. By targeting the entire network components of the near infiltration zone and partially resecting more distant infiltrative areas, supramarginal resection may offer a survival benefit compared to GTR, and several clinical analyses have supported this hypothesis [10, 11]. Existing scientific investigations on supramarginal resection, however, remain limited to retrospective studies [9, 12], frequently relying on case series from individual neurosurgical centers [11–14]. Consequently, despite promising retrospective data, the overall value of supramarginal resection in glioblastoma surgery remains unclear.

Additionally, supramarginal resection is not universally applicable to all tumor locations due to the potential for increased neurological morbidity when resecting brain tissue beyond the contrast-enhancing tumor mass [8]. These considerations present significant challenges in advancing research on supramarginal resection, particularly in designing prospective clinical trials to compare its outcomes with those of conventional GTR [15].

**Fig. 1:**
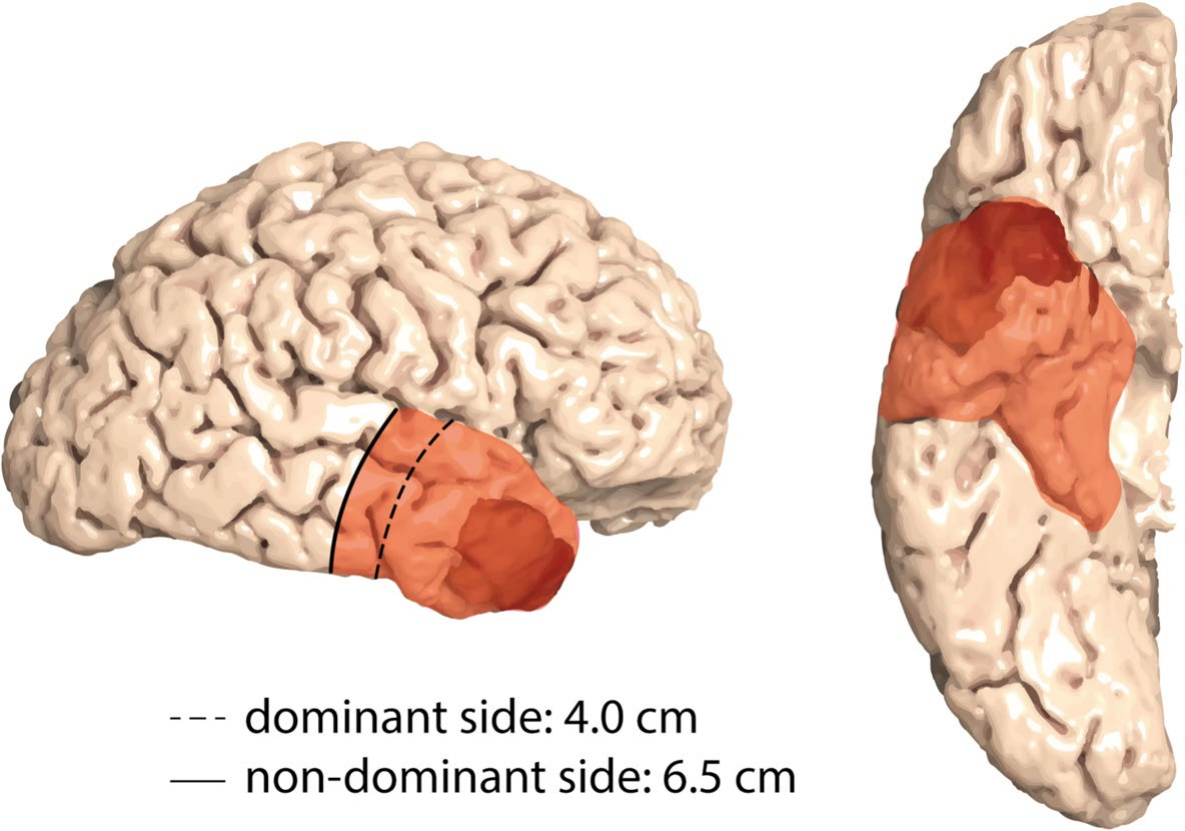
The ATL as a surgical approach for anterior temporal lobe glioblastoma. The adaptation of ATL from epilepsy surgery to glioblastoma surgery enables the resection of both the tumor bulk (depicted in dark red for this example) and extensive surrounding infiltrative tissue as an *in-toto* specimen. Scheme modified from Wiebe et al. [18]. ATL, anterior temporal lobectomy

One strategy within supramarginal resection is the so-called lobectomy, in which the entirety or a defined portion of one of the four brain lobes is removed [16, 17]. Besides to frontal lobectomy, which involves the removal of large parts of the frontal lobe, anterior temporal lobectomy (ATL) is the most common variant. ATL is a highly standardized technique commonly employed in epilepsy surgery for patients with pharmacoresistant temporal lobe epilepsy across all ages [18, 19]. Its surgical methodology and postoperative outcomes, including neuropsychological effects associated with temporomesial resection, have been extensively studied [20–23]. Given its well-defined resection boundaries [18, 19] and demonstrated procedural safety [24], translating the ATL approach from epilepsy surgery to oncological neurosurgery provides a unique opportunity to evaluate the potential superiority of supramarginal resection over conventional GTR for glioblastomas located in the anterior temporal lobe (Fig. 1) [16, 25, 26].

Here, we present the protocol for a prospective, randomized phase III surgical trial, ATLAS/NOA-29, fully titled *Anterior temporal lobectomy versus gross-total resection in newly-diagnosed temporal lobe glioblastoma,* designed to evaluate the efficacy of supramarginal resection, with ATL serving as a paradigm for this approach in glioblastomas located in the anterior temporal lobe. Demonstrating the superiority of ATL over GTR could establish ATL as the standard surgical approach for isolated temporal glioblastomas while offering compelling evidence to support the broader applicability of supramarginal resection to other tumor locations.

## Methods/design

### Trial design

**Fig. 2:**
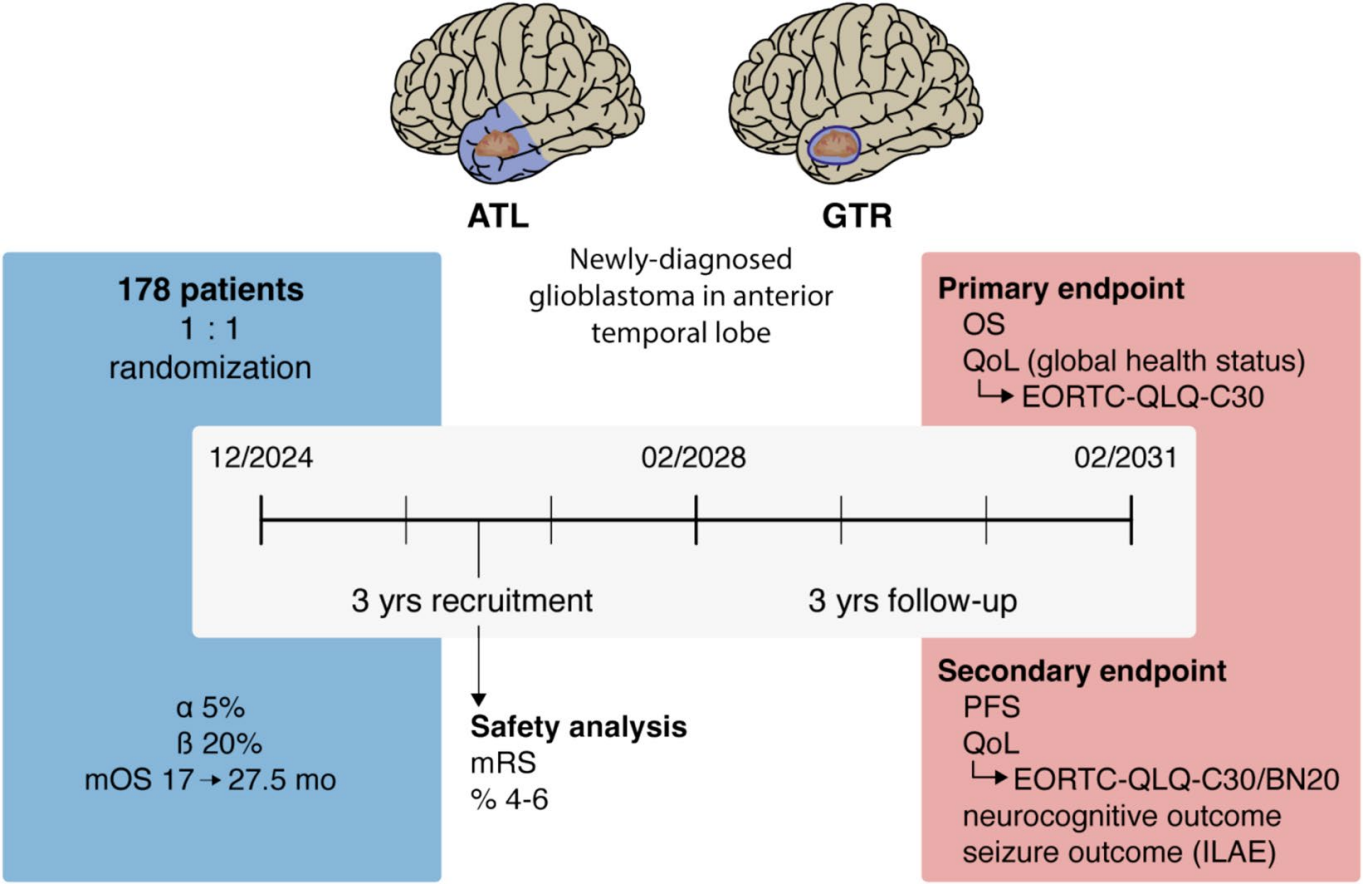
Graphical synopsis of the ATLAS/NOA-29 trial. ATL, anterior temporal lobectomy; EORTC, European Organization for Research and Treatment of Cancer; GTR, gross-total resection; ILAE, International League Against Epilepsy; QoL, quality of life; mo, months; mOS, median overall survival; mRS, modified Rankin Scale; PFS, progression-free survival; QLQ-C30/BN20, quality of life questionnaire modules; yrs, years

**Fig. 3:**
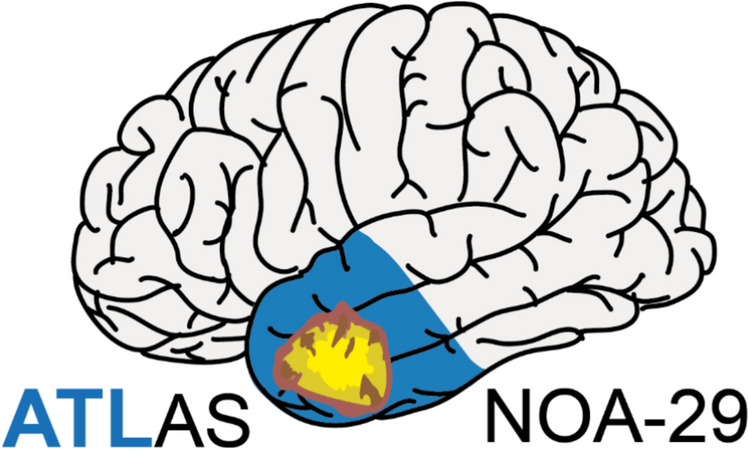
ATLAS/NOA-29 trial logo

The ATLAS/NOA-29 trial (Figs. 2 and 3) is an investigator-initiated, multicenter, multinational, prospective, phase III randomized (1:1) standard treatment-controlled study in patients with newly-diagnosed glioblastoma confined to the anterior temporal lobe.

### Objectives

The primary objective of this trial is to establish the superiority of ATL over GTR regarding OS and quality of life (QoL). Superiority will be demonstrated if ATL shows a significant improvement in OS while maintaining non-inferiority in QoL. Secondary objectives include progression-free survival (PFS), QoL, Karnofsky performance scale (KPS), modified Rankin Scale (mRS), neurocognitive functioning, and post-interventional seizure outcomes.

### Endpoints

The primary endpoint is OS in the modified intention-to-treat (mITT) population, defined as all randomized patients with histologically confirmed glioblastoma (CNS world health organization (WHO) grade 4, isocitrate dehydrogenase (IDH)-wildtype (wt)). If significant differences in OS are observed, the patient-reported QoL domain "global health status" from the European organization for research and treatment of cancer (EORTC) QLQ-C30 questionnaire will serve as a co-primary endpoint. Overall superiority of ATL requires a significant improvement in OS alongside non-inferiority in the development of global health status over time within the mITT population. Secondary endpoints include OS in the population of all patients randomized in the trial (intention-to-treat, ITT population) and in the per-protocol (PP) population. The QoL domain "global health status" from the EORTC QLQ-C30 questionnaire will also serve as a co-endpoint in these analyses, analogous to the primary endpoint. PFS, measured from the day of randomization until diagnosis of progressive disease based on MRI (RANO 2.0 criteria) [27], will be analyzed in the ITT, mITT, and PP populations. Separate OS and PFS analyses will also be conducted for patients with IDH-mutant gliomas. Further secondary endpoints will be evaluated in the mITT population and include QoL across six preferred domains of the EORTC QLQ-C30 and BN20 questionnaires (global health status, physical functioning, social functioning, cognitive functioning, communication deficit, and motor dysfunction), as well as all other domains of these questionnaires. Additional endpoints include PFS (as defined above), the KPS, the mRS, neurocognitive functioning assessed using the Verbaler Lern- und Merkfähigkeitstest (VLMT) [28, 29], the Boston Naming Test [30], and EpiTrack® [31], as well as seizure outcomes classified according to the International League Against Epilepsy (ILAE) [32].

Safety endpoints will be analyzed in the safety population, which includes all patients randomized in the trial. The mRS will serve as a key safety measure. In an interim safety analysis, the data safety monitoring board (DSMB) will evaluate whether the trial should continue if, six months after the inclusion of 57 patients, a clinically-relevant difference between treatment arms is observed in the rate of patients with mRS scores of 4–6 (assessed six months after each individual randomization). The mRS will also be monitored throughout the trial. Additional safety measures include periprocedural adverse events, assessed using patient safety indicators (PSIs) [33] and specific cranial-surgery-related complications (CSCs) [34] within 90 days of randomization, as well as adverse events (AEs) as per common terminology criteria for adverse events (CTCAE) version 5.0 observed within 30 days of randomization.

### Treatment groups

#### Experimental intervention

Patients in the experimental arm will undergo ATL as described by Wiebe et al. [18] (Fig. 1). ATL is a highly standardized surgical procedure commonly performed in patients with pharmacoresistant temporal lobe epilepsy [19]. The dorsal extent of the neocortical temporal lobe resection measures 6.5 cm from the temporal pole on the non-dominant side and 4.0 cm on the dominant side, as determined dorsally along the Sylvian fissure [18]. Language dominance is determined based on handedness (for details, see inclusion criteria). Typically, the neocortical portion can be resected as an *in-toto* specimen. The mesial resection includes the uncus of the temporal lobe, the amygdala, and the hippocampal head and body [19], as allocortical structures, usually extending to the level of the tectum or, at a minimum, to the lateral sulcus of the mesencephalon [35]. Standard practice involves resecting the hippocampal body together with the surrounding parahippocampal gyrus [36]. The extent of resection will be verified using early postoperative MRI (T2/fluid-attenuated inversion recovery (FLAIR) and gadolinium-enhanced T1 sequence) within 72 h after surgery.

#### Control intervention

Patients in the control arm will undergo GTR, defined as the removal of 100% of tumor tissue as visualized on gadolinium-enhanced MRI [9]. The extent of resection will be confirmed using early postoperative MRI (T2/FLAIR and gadolinium-enhanced T1 sequences) within 72 h after surgery.

In both treatment arms, resection will be followed by guideline-conform adjuvant first-line therapy. Options include:

- Standard radiochemotherapy with temozolomide (TMZ) according to Stupp et al. [37].
- For elderly patients (> 70 years), hypofractionated radiotherapy (RT) with or without TMZ chemotherapy, based on O-6-methylguanine-DNA methyltransferase (MGMT) promoter methylation status [38].
- For elderly patients, monotherapy with either RT or standard TMZ chemotherapy alone [39].
- For patients < 70 years with MGMT promoter-methylated glioblastoma, RT combined with CCNU/TMZ according to CeTeG/NOA-09 [40].
- Addition of tumor-treating fields (TTFs) is possible [41].

Participation in another interventional study is not permitted until progression, determined according to the response assessment in neuro-oncology (RANO) 2.0 criteria [27].

### Number of subjects

A total of 178 subjects will be randomized in a 1:1 ratio between the experimental and control arms.

### Planned time schedule

The total duration of the trial is planned to be 75 months. The recruitment period will last 36 months, followed by a follow-up period of at least 36 months per patient with a follow-up examination every 12 weeks. An interim safety analysis will be conducted six months after the randomization of the 57th patient to evaluate differences in clinical performance, measured by the mRS, between the experimental and control intervention groups. Three months are allocated for this interim analysis. The timeline from first patient in to last patient out is, thus, expected to be 75 months.

### Inclusion criteria

- Suspected glioblastoma with contrast-enhancement in preoperative MRI
- Diffuse high-grade glioma in frozen section procedure, newly-diagnosed
- Tumor localization (in gadolinium-enhanced MRI): solely temporal, non-dominant side (right hemisphere in right-handed patients, or left-handed patients after testing for dominance): within 6.5 cm from the temporal pole; dominant side (left hemisphere in right-handed patients, all left-handed patients unless additional testing for dominance performed): within 4.0 cm from the temporal pole, as determined dorsally along the Sylvian fissure.
  - ▪ Explanation: In the case of right-handedness, the left hemisphere is considered the dominant hemisphere. For left-handed patients, the dominant hemisphere is uncertain. Consequently, the extent of resection for left-handed patients within the study should remain within 4.0 cm from the temporal pole for the ATL approach. If additional diagnostic procedures confirm the non-dominant hemisphere (e.g., Wada test, functional MRI, functional transcranial Doppler ultrasonography, magnetoencephalography, or awake mapping methods), the resection boundary may be extended to 6.5 cm at the discretion of the treating neurosurgeon.
- Macroscopic complete resection (no remaining contrast-enhancing tumoral lesion on early postoperative MRI) is achievable (decision of the treating neurosurgeon)
- In case further T1-contrast-enhancing and/or T2/FLAIR lesions are detected beyond the resection margins (6.5 cm on the non-dominant side and 4.0 cm on the dominant side), these lesions are not attributed to the tumor (except perifocal edema) but to other conditions according to the local treating neurosurgeon
  - ▪ Explanation: Vascular pathologies or other T1-contrast-enhancing lesions that are not attributable to the tumor beyond and inside the resection margins, as well as T2/FLAIR lesions attributable to peritumoral edema do not lead to the exclusion from the study. However, T1-contrast enhancing lesions beyond the resection margins that are attributable to the tumor and multifocal gliomas do lead to an exclusion from the study (for definition of multifocal glioma see 7.3.)
-  ≥ 18 and < 75 years of age
- KPS ≥ 70%
- Estimated life expectancy of at least 6 months
- Written informed consent
- Cognitive state to understand the rationale and necessity of the study therapy and procedures
- Patient compliance and geographic proximity that allow adequate follow-up
- For patients with childbearing potential: negative serum pregnancy test (beta-HCG) at baseline visit, patient’s commitment to use an approved contraceptive method during the trial and for 3 months after (Pearl index < 1%)
- Adequate organ function at baseline visit that does not preclude alkylating chemotherapy and neurosurgical procedures (all criteria required):
  - ▪ Adequate bone marrow reserve
    - white blood cell (WBC) count ≥ 3.000/µl
    - granulocyte count > 1.500/µl
    - platelets ≥ 100.000/µl
    - haemoglobin ≥ 10 g/dl
  - ▪ Adequate liver function
    - bilirubin < 1.5 times above upper limit of normal range (ULN)
    - alanine transaminase (ALT/SGPT) and aspartate transaminase (AST/ALAT) < 3 times ULN
  - ▪ Adequate renal function
    - creatinine < 1.5 times ULN
- Adequate blood clotting: PTT not exceeding the upper limit of normal range and INR < 1.5; in case of intake of anticoagulant medication or platelet function inhibitors, the coagulation analysis must show no detectable effect in specific blood tests (as described below) at the time of surgery, and discontinuation of the anticoagulant medication must be justifiable for at least 1 week postoperatively
  - ▪ Direct acting oral anticoagulants (e.g., rivaroxaban, apixaban, edoxaban, dabigatran): aFXa-activity (anti-factor Xa) within the normal range (rivaroxaban, apixaban, edoxaban), TT/TCT (thrombin clotting time) or ecarin clotting time (ECT) not exceeding the upper limit of normal range (Dabigatran) or verification of subtherapeutic drug levels (apixaban, edoxaban, rivaroxaban, dabigatran)
  - ▪ Vitamin K antagonists (coumarins): INR < 1.5
  - ▪ Unfractionated heparin (UFH) and argatroban: aPTT not exceeding the upper limit of normal range
  - ▪ Fractionated heparin/low-molecular-weight heparin (e.g., dalteparin, enoxaparin), heparinoid (e.g., fondaparinux, danaparoid): aFXa-activity within the normal range
  - ▪ Antiplatelet agents (aspirin, clopidogrel, prasugrel, ticagrelor): PFA (platelet function analyzer) test not exceeding the upper limit of normal range (aspirin), whole blood aggregometry not below lower limits of normal range (clopidogrel, prasugrel, ticagrelor)

### Exclusion criteria

- The dorsal extent of the gadolinium-enhancing tumor reaches more than 6.5 cm measured from the temporal pole dorsally along the Sylvian fissure on the non-dominant side or more than 4.0 cm on the dominant side
- The extent of GTR is projected to closely approximate that of ATL based on preoperative MRI findings (at the discretion of the treating neurosurgeon)
  - ▪ Explanation: This applies if the gadolinium-enhancing tumor components comprise ≥ 90% of the ATL volume, effectively making the extent of GTR equivalent to that of ATL
- Temporal tumor with gadolinium-enhancing infiltration of further lobi and/or multifocal tumor
  - ▪ Explanation: Multifocal tumor is defined as two or more lesions within the same hemisphere but separated by white matter tracts
- Prior malignancy (unless adequately treated carcinoma in situ of the cervix or nonmelanoma skin cancer), unless the prior malignancy was diagnosed and definitively treated at least 5 years previously with no subsequent evidence of recurrence
- Prior chemotherapy, systemic or local treatment with DNA-damaging agents, tyrosine kinase inhibitors or anti-angiogenic agents for any cancer
- Prior RT to the brain
- Active infection and infections preventing surgery and/or further chemotherapy (at the discretion of the treating neurosurgeon)
- Conditions with increased risk for intraoperative and/or perioperative bleeding
  - ▪ Explanation: Patients with pro- or anticoagulant coagulation disorders may participate in the study if, after consultation with a coagulation specialist or at the discretion of the neurosurgeon, the coagulation status has been optimized. Study participation is not possible for patients on anticoagulation or antiplatelet therapy if the medication cannot be discontinued prior to surgery and at least 1 week postoperatively (e.g., for high cardiovascular risk patients), or the relevant coagulation analysis (as indicated in the inclusion criteria) does not indicate normal coagulation status at time of surgery
- Female patients that are pregnant or breastfeeding or patients with childbearing potential that do not commit to use an approved contraceptive method during the trial and for 3 months after (Pearl index < 1%)
- History of disease with poor prognosis (e.g., severe heart failure) with an estimated life-expectancy < 6 months
- Unable to undergo contrast-enhanced MRI
- Any psychological, cognitive, familial, sociological or geographical condition potentially hampering compliance with the study protocol and follow-up scheduled visits (at the discretion of the investigator)
- Patients not capable of giving consent
- Patients incapacitated and unable to understand the nature, scope, significance and consequence of this clinical trial

### Gender distribution

While some studies have suggested a potential prognostic role for gender in glioblastoma, with better survival observed in females compared to males [1, 42] recent analyses of large real-world data indicate that this difference disappears after adjusting for known prognostic parameters like the *MGMT* promoter methylation status as well as the chosen treatment modalities [43]. Moreover, according to major clinical guidelines, such as those from the National Comprehensive Cancer Network (NCCN) and the European Association for Neuro-Oncology (EANO) [44], gender is not considered a universally recognized prognostic factor in glioblastoma. Therefore, no specific gender ratio has been specified for this study.

### Randomization

A unique feature of the ATLAS/NOA-29 trial is the intraoperative randomization process. Patients with imaging evidence suggestive of newly-diagnosed glioblastoma confined to the anterior temporal lobe will undergo craniotomy, typically performed as a pterional or temporal craniotomy, to allow for both temporal GTR and ATL. Once the dura is opened and tumor access is achieved, tumor tissue will be sent for frozen section analysis by the neuropathologist or pathologist in charge. Randomization will only occur after the (neuro)-pathologist has confirmed the diagnosis of diffuse high-grade glioma based on the frozen section results. The frozen section procedure, as performed in this study, is expected to prolong the surgery by approximately 10 to 20 min.

Randomization will be performed using the method of permuted blocks with variable block sizes. The randomization number will be assigned through the electronic case report form (eCRF) system, in a 1:1 ratio between the experimental (ATL) and control (GTR) interventions. Stratification will occur based on two criteria: age group (< 50 years or ≥ 50 years) and pre-operative KPS (70–80% vs. 90–100%).

Blinding of the surgical procedure is not feasible due to its inherent nature. The operating neurosurgeon and other treating physicians must review MRI scans during follow-up examinations, which will reveal the surgical approach. Consequently, the randomization process must be open.

### Time schedule of measurements

**Fig. 4:**
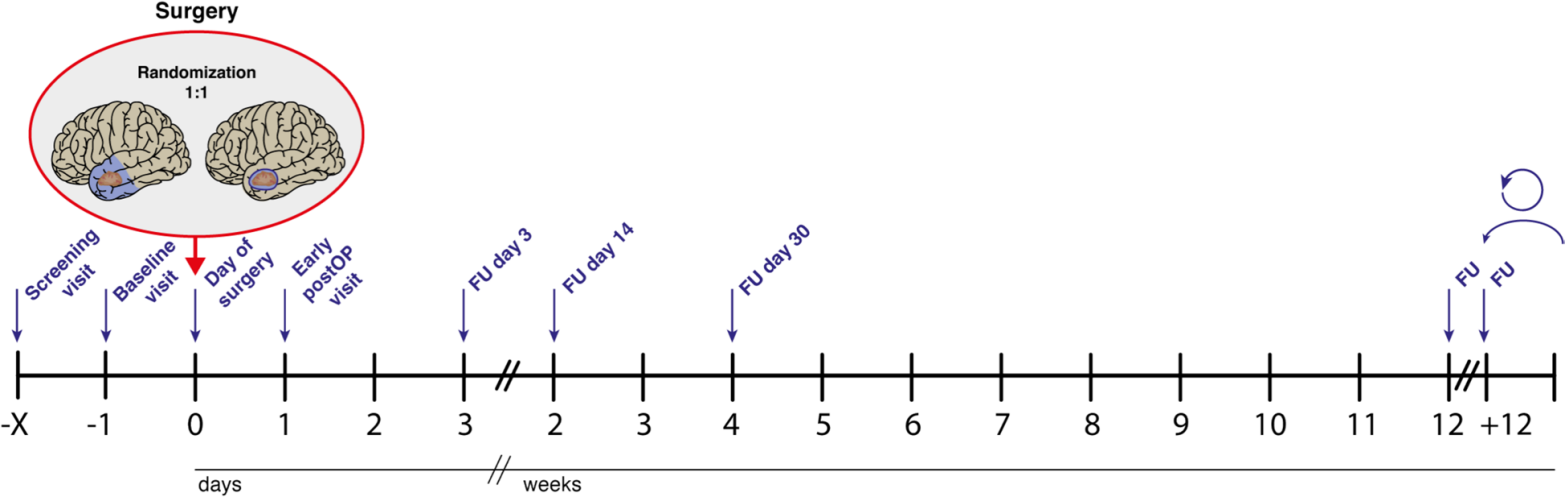
Overview of study visits. FU, follow-up

The schedule of activities (Fig. 4 and Table 1) includes a Screening visit (Visit 1) on the day of admission to the hospital, typically 1–2 days prior to surgery, but no earlier than two weeks before surgery. During this visit, patients will be screened for inclusion and exclusion criteria. Visit 2 (Baseline visit) involves obtaining written informed consent for trial participation. Visit 3 (Day 0), the day of surgery, includes the randomization process, which will only be performed after the diagnosis of diffuse high-grade glioma is confirmed via frozen section procedure. Furthermore, surgery-related data will be collected during this visit, including any intraoperative complications specifically related to temporomesial anatomy.

**Table 1:** Schedule of activities

Activities	Screening	Baseline	Day of Surgery	Early PostOP	FU d3	FU d14	FU d30	FU every 12w
I/E criteria	✔	-	-	-	-	-	-	-
Demographics, medical history	✔	-	-	-	-	-	-	-
Written informed consent	-	✔	-	-	-	-	-	-
Vital signs (heart frequency, blood pressure, body temperature), weight	-	✔	-	✔	✔	✔	✔	✔
Physical examination	-	✔	-	✔	✔	✔	✔	✔
Neurological examination (NANO scale)	-	✔	-	✔	✔	✔	✔	✔
Orthoptic examination	-	✔	-	-	-	✔	-	-
Seizure status (ILAE)	-	✔	✔	✔	✔	✔	✔	✔
Current medication (including steroids)	-	✔	✔	✔	✔	✔	✔	✔
ASA score	-	✔	-	-	-	-	-	-
KPS	-	✔	-	✔	✔	✔	✔	✔
MMSE	-	✔	-	-	-	✔	✔	✔
Modified Rankin Scale (mRS)	-	✔	-	✔	✔	✔	✔	✔
Neurocognitive testing (VLMT, Boston Naming test, EpiTrack ®)	-	✔	-	-	-	-	✔	✔
QoL questionnaire (EORTC-QLQ-C30 and BN20)	-	✔	-	-	-	✔	✔	✔ ^c^
Complete blood count	-	✔	-	✔	✔	-	-	-
Coagulation analysis (including preoperative tests as specified in 2.7)	-	✔	-	✔	-	-	-	-
Serum chemistry	-	✔	-	✔	✔	-	-	-
Pregnancy test ^b^	-	✔	-	-	-	-	-	-
Gadolinium-enhanced MRI	-	✔	-	-	✔	-	-	✔
EEG	-	✔	-	-	-	-	✔	-
Adverse events (CTCAE)	-	-	✔	✔	✔	✔	✔	-
Randomization	-	-	✔	-	-	-	-	-
Surgery-related data (IOM, duration, blood loss, intraoperative seizures etc.)	-	-	✔	-	-	-	-	-
Survival status ^a^	-	-	-	✔	✔	✔	✔	✔
Periprocedural adverse events (PSIs, CSCs)	-	-	-	✔	✔	✔	✔	✔ ^d^
Length of mechanical ventilation	-	-	-	✔	✔	✔	-	-
Histopathological results	-	-	-	-	-	-	✔	-
Length of hospital stay	-	-	-	-	-	✔	✔	✔
Radiotherapy (day of start, number of days, total Gy)	-	-	-	-	-	✔	✔	✔
Tumor-directed medical therapy (drug, dose, schedule)	-	-	-	-	-	✔	✔	✔
Other tumor-directed treatment (e.g. TTFs)	-	-	-	-	-	✔	✔	✔
Assessment of progression (RANO 2.0 criteria)	-	-	-	-	-	-	-	✔

*AS*, American society of anesthesiologists, *CSCs* cranial-surgery-related complications, *CTCAE* common terminology criteria for adverse events, *d* day, *EEG* electroencephalography, *EORTC* European organization for research and treatment of cancer, *FU* follow-up, *Gy* Gray, *I/E* inclusion/exclusion, *ILAE* International League Against Epilepsy, *KPS* Karnofsky performance scale, *MMSE* mini mental state examination, *PSIs* patient safety indicators, *QLQ* quality of life questionnaire, *RANO* response assessment in neuro-oncology, *VLMT* Verbaler Lern- und Merkfähigkeitstest

^a^ until end of the trial, i.e. 3 years after randomization of the last patient or until death

^b^ for women with childbearing potential

^c^ after day 90 visit (first 12 week FU): every 24 weeks

^d^ only until day 90

Postoperative visits are scheduled as follows: Visit 4 will occur on Day 1 (early postoperative), Visit 5 on Day 3 (postoperative), Visit 6 on Day 14, Visit 7 on Day 30, and Visit 8 on Day 90. Subsequent follow-up visits will be conducted every 12 weeks (± 5 days) until death. AEs will be assessed according to CTCAE5, starting from Visit 2 and continuing through Visit 7. Particular attention will be given to postoperative complications, which will be recorded from Visit 4 through Visit 8, with potential late-onset complications assessed during Visit 8.

Timing deviations from the defined schedule are considered protocol violations under certain conditions. For Visit 6, a deviation of more than 2 days is regarded as a violation, while for Visit 7 and all subsequent visits, deviations of more than 5 days are considered violations. No deviations are permitted for Visits 2 through 5.

### Methods of assessment

This section provides an overview and explanations of the examinations and procedures to be conducted in this trial. All data will be documented in the eCRF for indicated visits and follow-up examinations as outlined in the schedule of activities (Table 1).

#### Overall survival and progression-free survival

OS is defined as the time from the day of randomization/day of surgery to the respective patient’s death. Progression is defined as the first documented evidence of progressive disease according to the RANO 2.0 criteria [27]. PFS is measured from randomization at the day of surgery until progression or death.

#### Quality of life

The EORTC QoL questionnaire modules QLQ-C30 and BN20 will be used to evaluate the QoL of patients [45–47]. These well-validated and internationally recognized tools assess QoL across 26 domains. Based on prior studies [40, 48, 49], specific domains of particular relevance for brain tumor patients will be emphasized, including global health status, physical functioning, social functioning, cognitive functioning, communication deficit, and motor dysfunction. The questionnaire is designed to be completed within approximately 10 min in both the pretreatment phase and during ongoing treatment.

The questionnaire's validity has been demonstrated across various cancer populations and disease stages, including their respective treatments. Test–retest reliability, assessed within a four-day interval, has shown good to excellent consistency for all functional scales, with Pearson’s r values ranging from 0.72 to 0.91 [45–47, 49–51].

For this trial, patients will complete the questionnaires using tablets as electronic patient-reported outcomes (ePRO) devices. Study staff will assist patients in handling the ePRO instruments and ensure the completeness of responses before releasing the data for transfer. QoL assessments will be conducted at the baseline visit, on follow-up days 14 and 30, and at each 12-week visit.

#### Neurocognitive functioning

Neurocognitive function will be assessed using a test battery specifically designed to evaluate temporo-lateral, temporo-mesial, and frontal lobe functions, including memory, language, and executive functions. All tests have been validated for use in monitoring and outcome evaluation in temporal lobe epilepsy surgery [20, 21, 52, 53]. Cognitive functions across all domains will be quantitatively assessed preoperatively and postoperatively at Visit 7 (Day 30), Visit 8 (Day 90), and every 24 weeks until the conclusion of the trial. These assessments will focus on both group-level and individual-level changes over time.

Neurocognitive test battery consists of the VLMT (duration: 30 min including 20 min break between learning and retention) for verbal memory [28, 29], Boston Naming Test (duration: max. 5 min) for naming [30] and the EpiTrack® (duration: max. 15 min) for executive functions [31].

All chosen tests are standard for monitoring epilepsy surgery and temporal lobe surgery in particular [21]. The VLMT is a verbal learning and memory test that follows a list-learning paradigm with a 15-word list (A) to be learned over five learning trials with immediate recall, followed by learning a distractor list (B) once. This is followed by free recall of list A after distraction by learning and recalling list B and again after a delay. Finally, there is a recognition trial that requires identifying list A words from a list containing list A and B words, as well as new distractor words that are phonemically or semantically related to list A and B words. The test has proven its sensitivity to temporal lobe pathology and surgery in several studies [54–60].

The EpiTrack® is a short screening test for executive functions comprising subtests which assess psychomotor speed, mental flexibility, response inhibition, fluency, working memory, and anticipation. The EpiTrack® and its subtests are sensitive to frontal lobe functioning [61, 62] and to CNS drug effects and have been used to monitor epilepsy surgery [63, 64]. The Boston Naming test assesses naming and word finding in the language domain and is a standard procedure to monitor language functions in epilepsy surgery [65, 66].

The Boston Naming Test [30] and EpiTrack® [31] will be performed during the 20-min break of the VLMT [28, 29]. Consequently, the entire neurocognitive test battery will take no more than 30 min, ensuring it is well-tolerated by brain tumor patients. This test battery can be conducted by a study nurse and does not require the presence of a neuropsychologist.

The mini mental state examination (MMSE), a general test for neurocognitive functioning with a maximum score of 30 points [67], assesses various cognitive domains, including orientation, concentration, delayed recall, visual construction, and aphasic problems. The MMSE [67] will be performed at baseline, on follow-up days 14 and 30, and at each 12-week visit.

#### Seizure status

Tumor-associated seizures are defined as any unprovoked seizure occurrence with evidence of a concordant tumorous lesion in MRI examinations prior to surgery. During the baseline visit, seizure frequency will be assessed as the average number of seizures per month. Seizures will be classified based on their origin in the brain, the degree of awareness during the seizure, and the level of body movement, following the 2017 classification of the ILAE commission on classification and terminology [32]. Focal onset seizures will be categorized as focal aware (including the former term aura), focal impaired awareness, focal motor onset, focal non-motor onset, or focal to bilateral tonic–clonic. Generalized onset seizures will be categorized as generalized motor (tonic–clonic) or generalized non-motor (absence).

**Fig. 5:**
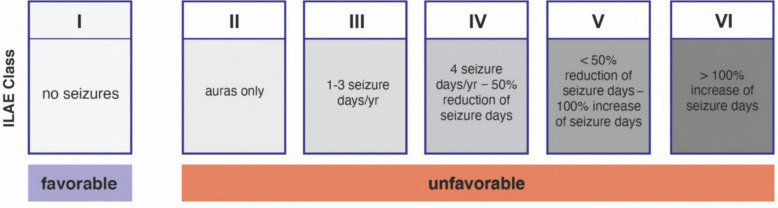
Graphical overview of the ILAE classification system for assessing postoperative seizure outcome. Scheme adapted from Wieser et al. [68]. ILAE, International League Against Epilepsy; yr, year

Postoperative seizure outcome will be analyzed based on seizure status at 12 weeks, 6 months, and each subsequent 12-week follow-up examination. Seizure outcomes will be classified according to the ILAE system used for epilepsy surgical procedures. Favorable seizure outcomes will be defined as seizure freedom (ILAE class 1), while unfavorable outcomes will include ILAE classes 2–6, covering pure auras, rare to no improvement, or worsening of seizure frequency [50]. Detailed information on the ILAE classification system for postoperative seizure outcome is presented in Fig. 5.

In addition to documenting seizure status according to the ILAE classification, electroencephalography (EEG) will be performed preoperatively during the baseline visit (Visit 2) and postoperatively at 30 days after surgery (Visit 7). EEG reports must note any pathological findings and specify whether these include epileptiform activity, status epilepticus, focal findings, or other abnormalities. Additional EEG recordings may be performed at any time but are not mandatory.

#### Perioperative complications

Postoperative complications will be assessed by PSIs [33] and specific CSCs as previously described [34].

PSIs introduced by the Agency of Healthcare and Quality [33] will entail pressure ulcer, iatrogenic pneumothorax, vascular catheter-related infection, transfusion reaction, retained surgical item, peri- and postoperative hemorrhage, acute postoperative respiratory failure, pulmonary embolism, deep venous thrombosis, postoperative sepsis, wound dehiscence, accidental puncture or laceration, postoperative hip fracture, postoperative physiologic and/or metabolic dearrangement. Peri- and postoperative hemorrhage will be defined as PSI in case they will require further surgical therapy.

CSCs will consist of postoperative ischemic infarction, cerebro-spinal fluid leakage, wound infection, atrophic wound healing disorder, postoperative meningitis and/or ventriculitis, emypema and postoperative permanent new or worsened neurological deficits including speech and language deficits [34]. Postoperative cerebrospinal fluid leakage will be defined as CSC if further surgical/interventional therapy (surgical wound examination, transient insertion of a lumbar drainage system and/or surgical insertion of a permanent shunt system) is required. Wound infection and postoperative meningitis will be defined as CSC, if further antibiotic-based and/or surgical therapy is needed.

PSIs and CSCs will be divided into early postoperative complications occurring within the time span of day 0 until day 30 (Visit 7) as previously described [34] and late postoperative events occurring from day 31 until day 90 (Visit 8).

The outcome will be documented as resolved, resolved with sequelae, ongoing at end of study or death.

Furthermore, iatrogenic damage of the middle cerebral artery (MCA) and/or its lenticulostriate branches, the anterior choroidal artery, and the P2 segment of the posterior cerebral artery (PCA) will be collected as intraoperative complications specifically related to temporomesial surgery.

#### Surgery-related data

Details of the surgical procedure will include the duration of the surgery, measured from incision to the completion of suturing, as well as the method of dural closure and whether a subgaleal drain was inserted. Intraoperative seizures, if any, must be documented, including their semiology, frequency, and the medications administered to manage them. Additionally, the amount of blood loss, the number of units of red cell concentrates and platelets transfused, and any clotting factors substituted must be recorded.

The use of adjunct technologies during surgery is optional and left to the discretion of the treating neurosurgeon. These include the administration of 5-ALA or other fluorescent dyes, the use of intraoperative MRI, and the performance of awake surgery.

Intraoperative neuromonitoring (IOM) will include the intraoperative recording of somatosensory evoked potentials (SEPs) and motor evoked potentials (MEPs). SEPs will be recorded following stimulation of the median nerve, with significant warning criteria defined as latency increases of > 10% or amplitude decreases of > 50%, whether transient or permanent [69, 70]. These changes must be documented in the eCRF.

For MEP measurements, subdermal needle placements will include the musculus abductor pollicis brevis (thenar), musculus adductor digiti minimi (hypothenar), and musculus tibialis anterior (lower extremity). A decline in MEP amplitude of > 50%, not attributable to technical issues, will be regarded as significant deterioration and must also be documented in the eCRF.

#### Orthoptic examination

An orthoptic examination, including the assessment of visual acuity and visual fields, will be conducted preoperatively during the baseline visit (Visit 2) and postoperatively 14 days after surgery (Visit 6). Any pathological findings must be documented in the examination report and specified in detail if observed.

#### Histopathological and molecular genetic results

The fresh frozen section procedure, mandatory for randomization, requires at least three of the four criteria defined by the WHO grading system to be met in support of the diagnosis of a diffuse high-grade glioma [71]. These criteria include high cellularity and mitotic activity, a diffuse infiltration pattern, microvascular proliferation, and necrosis.

Final histopathological findings confirming glioblastoma (CNS WHO grade 4), IDH wildtype, must be recorded in the eCRF. If other histological findings are identified that were classified as a diffuse high-grade glioma in the fresh frozen section procedure, they must be specified in the eCRF. Additionally, the MGMT promoter methylation status should be determined and categorized as hypermethylated (hypermethylation of the MGMT promoter), nonmethylated (no hypermethylation of the MGMT promoter), or not available/inconclusive. Optionally, any additional results from histopathological or molecular pathology analyses can also be entered into the eCRF.

#### MRI protocols

All MRI examinations have to include the following sequences:

- Pre-contrast injection: T2-w (e.g., turbo-spin-echo, TSE); T2-FLAIR; 3D T1-w (e.g., gradient-echo, GRE; or T1-w in two planes, one of them axial)
- Post-contrast injection: 3D T1-w (e.g., gradient-echo GRE; or T1-w in two planes, one of them axial)

Additional sequences are optional but can be included at the discretion of the treating physician.

For T2, FLAIR, DWI, and SWI/T2* sequences that are not performed in 3D, it is recommended to acquire the images in the axial plane to ensure structured and comparable evaluation. To ensure the reproducibility of MRI evaluation, all MRIs have to be reviewed post-hoc by the reference neuroradiologist Prof. Dr. Alexander Radbruch (Department of Neuroradiology, University Hospital Bonn). The MRIs will be sent blinded regarding the participating center (only labelled with the Randomization-ID) to the reference neuroradiologist to ensure an unbiased progression assessment.

Mandatory MRI examinations will be conducted as part of the standard treatment protocol. These include a preoperative MRI (baseline visit; any MRI performed within one week prior to surgery can be considered as the baseline MRI), an early postoperative MRI (performed within 72 h after surgery), and follow-up MRIs every three months after surgery. Additionally, a post-radiation MRI within four weeks of completing radiotherapy is recommended but not mandatory.

To evaluate protocol adherence regarding the surgical procedures in the experimental (ATL) and control (GTR) arms, the 72-h postoperative MRI will be reviewed by two experienced neurosurgeons. The resection results will be graded as follows:

- GTR: Resection cavity without remaining contrast-enhancing tissue, subtotal resection, or partial resection.
- ATL: Anatomical boundaries for ATL (as described above) fulfilled or not fulfilled.

#### Concomitant and postoperative treatment/medication

All tumor-directed therapies, including RT, chemotherapy, TTFs, and immunotherapy, must be documented from day 1 prior to surgery (baseline visit) until the end-of-study visit. Additionally, all CNS-active medications, such as psychotropic drugs, seizure-suppressing medications, and anti-edematous treatments (necessary for RANO 2.0 classification [27]), must be recorded in the eCRF for the same period. Anticoagulant and antibiotic therapies should be documented from day 1 prior to surgery until at least the day 90 visit. While the recording of other long-term medications, such as blood pressure medication, gastric protection, or dietary supplements, is desirable for the entire study duration, it is not mandatory.

#### Adverse events

All AEs must be documented in the eCRF using the respective Adverse Event Report Form. Systematic collection and documentation of AEs will be conducted from the time of randomization until Visit 7 (Day 30 postoperative).

PSIs [33] and CSCs [34] are not classified as AEs in this trial and will be documented separately in the eCRF.

#### Data safety monitoring board (DSMB)

The DSMB is an independent committee tasked with monitoring the progress of the study, ensuring the safety of trial participants, and assessing the quality of the collected data through monitoring reports. It is responsible for providing recommendations regarding the continuation, modification, or discontinuation of the trial. The DSMB will convene once per year during the recruitment phase to review data on AEs, PSIs, and CSCs. Additionally, six months after the randomization of the 57th patient, the DSMB will review the results of the interim safety analysis. Based on this review, the DSMB may propose modifications to the trial, such as stopping the trial or amending the protocol.

The DSMB will consist of at least three members with expertise in neurosurgery, neurology, and biometrics, as well as experience in the conduct of clinical trials. It is charged with reviewing safety data from both arms of the trial and is empowered to stop the trial if evidence of harm is observed, but it does not have the authority to terminate the trial based on a lack of efficacy. Furthermore, the DSMB will provide insights on emerging therapeutic or diagnostic advances during the course of the trial that could influence the outcomes. If protocol modifications are deemed necessary, close collaboration between the DSMB, the SZB staff, and the study leadership will be required.

## Statistics and analysis

### Sample size calculation

The sample size calculation is based on the primary endpoints of OS and QoL, with equal allocation of subjects to the two treatment groups. Assuming a two-sided alpha level of 5%, median OS times of 17 months in the control group and 27.5 months in the experimental group, a 3-year accrual period, and a 3-year follow-up period (resulting in a total trial duration of 72 months), a total of 170 subjects with a confirmed diagnosis of glioblastoma will provide 80% power to test the null hypothesis of equal OS between the two groups (two-sided log-rank test). The assumed survival benefit of a 10-month improvement with ATL over GTR is based on existing data from retrospective glioblastoma surgery studies involving ATL [16, 25] as well as analyses of EORTC datasets that are not specific to temporal tumor localization [9].

It is anticipated that approximately 4.5% of randomized patients will not have glioblastoma CNS WHO grade 4 but rather a different subtype of diffuse high-grade glioma, as determined by the final integrated histopathological and molecular diagnosis according to the 2021 WHO classification [55]. If the final diagnosis does not confirm glioblastoma IDH wildtype (CNS WHO grade 4), these patients will remain in the trial for safety follow-up but will be replaced in their respective groups and excluded from the mITT population. This replacement strategy will result in a maximum of 178 randomized patients. All randomized patients, regardless of diagnosis, will undergo regular follow-up as outlined in the trial protocol for safety monitoring.

The two primary endpoints will be analyzed in a hierarchical order, with QoL being tested for non-inferiority in the experimental group compared to the control group if the null hypothesis for OS is rejected. QoL measurements will be conducted quarterly, and a prior study demonstrated an approximately linear time dependence of the QoL (= global health) score on time [49].

A simulation was performed to assess the non-inferiority of the slope of QoL scores over time in the two treatment groups, with a non-inferiority margin of 0.4 score points (using the quarter as the time unit). The simulation incorporated survival status based on the previously stated assumptions and assumed a compound symmetry for the correlation structure of QoL scores across time points, with a correlation coefficient of 0.7. The results indicate that a total of 170 evaluable subjects in the mITT population will provide approximately 80% power to test for non-inferiority of QoL (linear mixed-effects model with QoL score as dependent variable, random patient intercept, and treatment group + time as independent variables, 95% confidence interval of the interaction between time and treatment, 1000 Monte Carlo replications, mean power 83%, Monte Carlo error 1.2%).

### Definition of populations included in the analysis

The analysis of the primary endpoint will be performed on the mITT population comprising all randomized patients with glioblastoma IDHwt. Subjects will be analysed in the study arms to which they were randomized, irrespective of protocol violations.

The ITT population of all randomized patients (incl. the patients with IDHmut diffuse high-grade glioma) will be used for sensitivity analyses of survival, QoL-endpoints, mRS and safety analyses. The ITT population is identical to the safety population.

If subjects undergo surgical treatment with an intervention different from the one assigned according to the randomization schedule, safety analyses will be conducted based on the intervention actually received ("as treated" analysis) rather than the assigned randomization group.

The PP population is a subset of the mITT population and is defined as the group of patients with a glioblastoma IDHwt who had no major protocol violations, and underwent the examinations required for the assessment of the endpoints at predefined times. The PP population will be used for sensitivity analyses.

Additionally, a separate exploratory analysis of OS, PFS, and QoL endpoints will be conducted for the subgroup of patients with IDH-mutant diffuse high-grade glioma, expected to comprise approximately 4.5% of the study population.

### Primary outcome

The statistical analysis will be conducted at the Study Center Bonn (SZB) of the University of Bonn Medical Center under the direction of Prof. Dr. Matthias Schmid. The primary, confirmatory analysis will compare OS between the treatment groups using a two-sided log-rank test at a significance level of 5%. This analysis will be performed on the mITT population. The log-rank test will be stratified by age group (< 50 years or ≥ 50 years) and pre-operative KPS class (70–80% vs. 90–100%). Surviving patients and those lost to follow-up will be censored at the respective time points. OS will be summarized using Kaplan–Meier curves, with median survival estimates and corresponding 95% confidence intervals reported.

A sensitivity analysis for OS will be conducted using a Cox proportional hazards model with treatment group and MGMT promoter methylation status (hypermethylated vs. unmethylated) as independent variables. If the MGMT promoter methylation status is not available, missing data for these patients will be imputed. In another sensitivity analysis for OS, a Cox proportional hazards model with treatment group and center recruitment volume (high vs. low) will be fitted. Additionally, potential prognostic factors, including age, preoperative KPS, MGMT promoter methylation status (hypermethylated vs. unmethylated), and center recruitment volume (high vs. low), will be analyzed using a multivariate Cox proportional hazard model for OS.

If OS of ATL significantly differs from that of GTR, the patient-reported QoL domain "global health status" from the EORTC QLQ-C30 questionnaire will be analyzed as a co-primary endpoint. Changes from baseline in the experimental arm (ATL) will be compared to the standard arm (GTR) to assess non-inferiority, using a non-inferiority margin of 1.6 score points per year. This evaluation will be performed on the mITT population and using a linear mixed-effects model (QoL score as dependent variable, random patient intercept, treatment group + time as independent variables, estimating a 95% confidence interval of the interaction between time and treatment).

Overall superiority of ATL will require significantly prolonged OS alongside non-inferiority in the development of global health status over time. Due to the hierarchical testing approach, no adjustment of the type I error rate is necessary.

The above analyses will be repeated as a sensitivity analysis using the ITT population of all randomized patients, the PP population, and, exploratorily, the population of patients with IDHmut diffuse high-grade glioma. Any relevant differences between the results of the mITT, ITT and PP analyses will be critically discussed.

### Secondary outcome variables

Secondary endpoints will be analyzed descriptively in the mITT population, if not otherwise stated. QoL will be assessed using the standardized and validated EORTC QLQ-C30 and QLQ-BN20 questionnaires. These questionnaires include health-related QoL questions scored on a nominal scale (primarily values from 1 to 4). The responses to individual questions will be recorded for analysis, and questions related to the same QoL dimension (e.g., emotional well-being) will be aggregated to generate an overall score for that dimension, following the scoring manual.

The scores for different QoL dimensions in each treatment group will be analyzed descriptively based on repeated assessments throughout the trial. Analogously to the analysis of the co-primary endpoint, linear mixed-effects models will be applied to the data to evaluate potential differences in the trends of QoL measurements over time between the treatment groups.

PFS will be summarized in the same manner as OS. Median PFS time estimates and associated 95% confidence intervals will be reported. The analysis of PFS will also be performed, similarly to the primary endpoints, within the ITT- and PP-populations as well as in the small population of patients with IDHmut diffuse high-grade glioma.

Neurocognitive function will be analyzed on both the group and the individual level. Group-level analyses will be conducted using multivariable linear mixed-effects models with post hoc tests to evaluate group differences. The side of surgery (left/right) will serve as a primary independent variable alongside the study arm.

At the individual level, both objective and subjective test data will be analyzed based on categories of impairment relative to test norms at each time point. Changes will be considered individually significant if they indicate a shift between performance categories across the three assessments (*p* < 0.1).

### Safety analysis

All safety evaluations, including the analysis of mRS scores at six months post-randomization, will be conducted on the safety population, comprising all patients included in the trial. AE incidence will be summarized in tabular form, both for overall incidence and individual occurrence.

### Protocol violations

Protocol violations are defined as any deviations from the procedures outlined in this protocol, including missed evaluations, incorrect timing of evaluations as well as any non-adherence to the protocol that impacts the subject’s rights, safety, or welfare.

Once a subject is enrolled, it is the investigator’s responsibility to make reasonable efforts to correct any protocol violations and to ensure the subject's continued participation in the trial, if feasible. Protocol violations alone do not constitute sufficient justification for withdrawing a subject from the trial.

Protocol violations will be reported to the project leaders during the trial via monitoring reports. A comprehensive list of all protocol violations will be prepared, and their potential impact on the evaluation of the affected subjects will be critically discussed prior to statistical analysis.

### Handling of drop-outs, withdrawal, and missing data

Subjects who drop out of the trial prior to randomization will be categorized as screening failures, with the reasons for drop-out documented in a comprehensive list.

Subjects who drop out of the trial after randomization will be analyzed using all available data, except for those replaced due to a disconfirming diagnosis of glioblastoma. These subjects will be excluded from the efficacy analysis.

### Interim safety analysis

No interim efficacy analysis is planned for this trial. The DSMB will evaluate safety data from the trial and will convene at least once per year during the recruitment phase. Additionally, to ensure that the postoperative clinical status in the experimental (ATL) arm is not substantially worse compared to the standard (GTR) arm, an interim safety analysis will be conducted six months after the randomization of the 57th patient. This ensures that one-third of the planned trial population is evaluable for safety within the first six months of the trial.

For safety determination, the mRS score for each patient will be assessed six months after randomization. The rates of patients with an mRS score of 4 or higher (dichotomized endpoint) will be compared between the treatment arms.

## Monitoring and audits

Quality control and assurance during the ATLAS/NOA-29 trial will be conducted through monitoring and audits by representatives of the Clinical Study Core Unit, Study Center Bonn (SZB).

Monitoring ensures accurate and reliable data by assessing trial progress, compliance, and documentation accuracy. The monitor will review source documents, eCRFs, and facilities, and ensure adherence to protocols and AE documentation. The frequency and scope of monitoring visits are defined in the Monitoring Plan. Source Data Verification (SDV) will confirm the accuracy of CRF entries by comparing them with source data as outlined in the Monitoring Manual.

Audits may be conducted by SZB representatives to ensure compliance with ICH-GCP and trial protocols. Investigators must provide access to relevant documents and resolve any audit findings. Audit certificates will be included in the final study report.

## Accompanying scientific research program

In the ATL arm, tumor resection provides extensive tissue samples from different zones of the tumor. These range from the necrotic center and bulky tumor regions to infiltration zones, including areas without visible signs of infiltration on conventional MRI. The ATL procedure offers a unique opportunity to map cellular components, functional states, and network interactions across the tumor’s infiltration zones.

The tissue collected during ATL will be analyzed to correlate histological tumor and microenvironmental structures with MRI parameters across different zones of the resected temporal lobe gliomas. Comprehensive gene expression analysis of these zones will be conducted using single-cell RNA sequencing and spatial transcriptomics. Spatial deep phenotyping of neuronal, immune, and tumor cell compartments will be performed using multiplexed immunofluorescence (MIF) [72]. Additionally, large-scale network activity and excitability in centimeter-sized samples of temporal glioblastomas will be investigated. Findings from histological, molecular, and electrophysiological analyses will be correlated with patient survival and their response or resistance to treatment.

A biobank of human glioblastoma organoids (GBOs) will be established using samples derived from geographically distinct regions within the parental tumors and more distant tumor microenvironmental areas from patients undergoing the ATL approach. These organoids will be analyzed to reconstruct three-dimensional network arrangements based on tumor microtubes, enabling the delineation of the spatially variable dynamic makeup of network morphology in glioblastoma. Pharmacological testing will subsequently be conducted to investigate the effects of novel therapeutic approaches as a function of the spatial heterogeneity of malignant networks.

## Discussion

Supramarginal resection has emerged as a promising surgical approach to address the extensive infiltrative nature of glioblastomas [9, 26]. The broader extent of resection, with its inherent risk of neurological morbidity, confines its application to carefully selected tumor locations and extensions [8]. These constraints present significant challenges in designing prospective, randomized clinical trials aimed at moving beyond the current reliance on retrospective data and providing robust, high-quality evidence. The ATLAS/NOA-29 trial addresses this gap by selecting the temporal tumor location with an established surgical approach as a model to evaluate the clinical feasibility and overall value of supramarginal resection.

The rationale underpinning the ATLAS/NOA-29 trial stems from retrospective studies suggesting that ATL may offer significantly prolonged PFS and OS in patients with temporal lobe glioblastoma compared to conventional GTR [16, 25]. Additionally, retrospective data indicate that ATL maintains perioperative safety profiles comparable to those of GTR [34], while offering the added benefit of significantly improved postoperative seizure outcomes [73]. Building on the established use of ATL in epilepsy surgery [18], where the procedure involves resection of the amygdala and the anterior part of the hippocampus [19, 36], neurocognitive outcomes are a critical consideration when translating this approach to oncological neurosurgery. In epilepsy surgery, the neurocognitive effects of ATL have been extensively studied, revealing frequent impairments in visual and verbal memory functions, with manifestations differing by the side of resection [22]. In contrast, visuospatial abilities are generally preserved [22, 23]. Despite these potential deficits, ATL has been shown to maintain a high level of overall postoperative cognitive performance in patients with temporal lobe epilepsy [20, 23]. This evidence suggests that adapting ATL to the neurooncological context may allow for sufficient preservation of postoperative neurocognitive capacities while leveraging its benefits as a supramarginal resection strategy. In contrast to patients undergoing ATL for epilepsy, where compensatory reorganization of neurocognitive functions has been reported in both non-surgically and surgically treated cohorts [74–76], such adaptation is unlikely in temporal glioblastoma due to the inherently distinct disease dynamics of epilepsy and glioblastoma. Despite this, data from glioblastoma surgery demonstrate that functional status, as measured by the KPS, remains comparable between ATL and GTR [25]. While minor neuropsychological deficits may occur following the ATL approach, neurological and cognitive functions appear to be preserved at a level that supports a good QoL and facilitates daily activities, aligning with outcomes observed for GTR.

In light of its potential to prolong OS while largely preserving QoL, ATL as a supramarginal resection strategy emerges as a promising and viable neurosurgical approach for temporal lobe glioblastoma. The potential survival benefit, however, must be carefully balanced against the risk of neuropsychological and QoL impairments to assess its overall clinical value. A definitive evaluation of the net clinical benefit of ATL in temporal glioblastoma requires a randomized trial directly comparing ATL with standard GTR, using survival and QoL as co-primary endpoints, as outlined in the ATLAS/NOA-29 trial protocol.

## Conclusions

Integrating insights from epilepsy surgery into brain tumor surgery provides a valuable opportunity to apply decades of established knowledge to advance oncological neurosurgery. The adaptation of ATL, a highly standardized and well-defined procedure, offers a robust model for evaluating the potential benefits of supramarginal resection in glioblastoma surgery. Demonstrating the superiority of ATL over GTR in the ATLAS/NOA-29 trial could define ATL as the preferred surgical approach for isolated temporal glioblastoma and provide compelling evidence supporting the broader implementation of supramarginal resection in glioblastoma management.

## Data Availability

After the final publication of the trial results, the datasets used and/or analyzed during the trial will be made available upon reasonable request. The ATLAS/NOA-29 trial database is managed and controlled by the Study Center Bonn (SZB) and utilizes an eCRF within the REDCap EDC system. All trial data are securely documented and stored electronically in compliance with regulatory requirements. Following database closure, statisticians at the SZB will independently perform the analyses outlined in the Scientific Analysis Plan, without interference from the principal investigator. The primary endpoint analyses will be conducted by two independent statisticians to ensure objectivity and reliability.

## Abbreviations

AE: Adverse event
aFXa: Anti-factor Xa
ASA: American society of anesthesiologists
ATL: Anterior temporal lobectomy
CCNU: Lomustine
CE: Contrast-enhancing
CSCs: Cranial-surgery-related complications
CRF: Case report form
CSF: Cerebrospinal fluid
CTCEA: Common terminology criteria for adverse events
DSMB: Data safety monitoring board
eCRF: Electronic case report form
EDC: Electronic data capture
EEG: Electroencephalography
EORTC: European organization for research and treatment of cancer
FLAIR: Fluid-attenuated inversion recovery
FU: Follow-up
GBO: Glioblastoma organoids
GCP: Good clinical practice
GTR: Gross-total resection
Gy: Gray
ICH: International conference on harmonization
IDH: Isocitrate dehydrogenase
IIT: Investigator-initiated trial
ILAE: International League Against Epilepsy
ISF: Investigator site file
ITT: Intention-to-treat
KPS: Karnofsky performance scale
MCA: Middle cerebral artery
MGMT: O-6-methylguanine-DNA methyltransferase
MMSE: Mini mental state examination
MEPs: Motor evoked potentials
MIF: Multiplexed immunofluorescence
MRI: Magnetic resonance imaging
mRS: Modified Rankin Scale
OS: Overall survival
PCA: Posterior cerebral artery
PD: Progressive disease
PFA: Platelet function analyzer
PFS: Progression-free survival
PP: Per-protocol
PSIs: Patient safety indicators
QLQ: Quality of life questionnaire
QoL: Quality of Life
RANO: Response assessment in neuro-oncology
RT: Radiotherapy
SDV: Source data verification
SEPs: Somatosensory evoked potentials
SZB: Study center Bonn (Studienzentrum Bonn)
TMZ: Temozolomide
TTFs: Tumor-treating fields
ULN: Upper limit of normal range
VLMT: Verbaler Lern- und Merkfähigkeitstest
WBC: White blood cells
Wt: Wild type
